# Functional motifs in food webs and networks

**DOI:** 10.1073/pnas.2521927123

**Published:** 2026-01-29

**Authors:** Melanie Habermann, Ashkaan K. Fahimipour, Justin D. Yeakel, Thilo Gross

**Affiliations:** ^a^Biodiversity Theory, Helmholtz Institute for Functional Marine Biodiversity, Oldenburg 26129, Germany; ^b^Alfred-Wegener Institute, Bremerhaven 27075, Germany; ^c^Carl-von-Ossietzky University, Institute for Chemistry and Biology of the Marine Environment, Oldenburg 26129, Germany; ^d^Department of Biological Sciences and the Center for Complex Systems, Florida Atlantic University, Boca Raton, FL 33431; ^e^School of Natural Sciences, University of California, Merced, CA 95343; ^f^The Santa Fe Institute, Santa Fe, NM 87501

**Keywords:** functional motifs, reactivity, stability, food webs, networks

## Abstract

When studying a complex system, it is often useful to think of the system as a network of interacting units. One can then ask if some properties of the entire network are already explained by a small part of the network, a network motif. A famous example of an ecological motif is exploitative competition in food webs, where the presence of two species competing for a shared resource precludes the existence of a stable equilibrium for the whole system. However, other examples of motifs with such direct impacts on stability are not known. Here, we show why small motifs that allow conclusions on systemic stability are rare. More importantly, we show that another dynamical property, reactivity, is typically rooted in motifs. Computing the reactivity of motifs can reveal which parts of a network are prone to respond violently to perturbations. This highlights motif reactivity as a useful property to measure in real-world systems to understand likely modes of systemic failure in food webs or other networks in epidemics, supply chains, or power grids.

## Significance

Understanding how complex systems respond to disturbances and which parts of a system could respond violently when perturbed is a major challenge across disciplines. Using food webs as an example, we show that a system’s immediate response to disturbance, reactivity, is often caused by small motifs within the network. In ecology, where network data are scarce, this enables identification of subgroups of interacting species that pose the greatest risk. This approach also applies to other complex networks, from detecting risky friend groups in epidemics to tracing local origins of cascading failures in power grids.

In its modern form, the competitive exclusion principle states that the number of species coexisting in an ecological system cannot exceed the number of realized niches (1, 2). Phrased thus, the principle is tautological (3), as a niche is defined as circumstances that allow a species to persist. This tautological nature makes the competitive exclusion principle an inviolable law, whose apparent violation in nature has guided the search for overlooked niches (4–6), leading to major discoveries (4, 7–9).

The success of the competitive exclusion principle is rooted in its applicability to small, isolated parts of an ecological network, avoiding the complexity that exists in the wider system. The simplest case is the exploitative competition motif, where two unregulated consumers specialize on one resource (10). Hence, the presence of this motif in a food web implies that some (perhaps so-far undetected) internal regulation of the consumers must be present (5, 11). Importantly, this result is independent of the structure of the rest of the network, which makes the principle a valuable tool for ecology. It is thus reasonable to ask if there are other network motifs with similar systemic implications, which could lead to new principles as powerful as competitive exclusion. Although stable motifs have a tendency to stabilize food webs (12–15), even an extensive numerical search has not identified other motifs that have as clear-cut implications as the exploitative competition motif (16).

Here we use the term functional motif to indicate a part of the network that by its mere presence has implications that cannot be negated by the rest of the network. We explain why exploitative competition is a functional motif with respect to stability and also show why other such motifs are unlikely to exist. We then discuss other mechanisms by which motifs can become functional in any type of network. This leads to a discussion of reactivity, a different notion of ecological stability (17), for which we show that every subgraph of a network is a functional reactivity motif. If high reactivity is found in a part of the network, it will respond violently to disturbances in a way that cannot be compensated by the rest of the network. This highlights the reactivity of parts of a system as an important property that should be measured in real-world networks.

## Functional Stability Motifs

Consider a many-variable dynamical system whose dynamics are described by a system of differential equations. We can picture such a system as a network, where nodes correspond to variables and weighted directed links indicate the interaction between pairs of variables.

A common notion of stability, locally asymptotic stability, can be computed by studying so-called eigensolutions of the dynamics in the proximity of a dynamical behavior (18). In the simplest case, the behavior under consideration is stationarity in a steady state. The dynamics in the proximity are then captured by the Jacobian matrix, **J**, and the eigensolutions are given by the eigenvectors and eigenvalues, found by solving the eigenvalue equation

[1] $$\begin{array}{c} Jv=\lambda v, \end{array}$$

where v is an eigenvector and λ the corresponding eigenvalue. Specifically, a stationary state is stable if all λ have negative real parts. Similar conditions also exist for nonstationary states but are in practice harder to compute and hence less frequently evaluated.

Since the presence of a single nonnegative Jacobian eigenvalue makes a system unstable, a part is a functional stability motif if its presence in a system implies that at least one eigenvalue will have a nonnegative real part.

There are two different ways in which the presence of a specific motif can imply dynamical instability: First, the presence of a motif could stipulate that there must be a specific eigenvalue. Second, a given motif could show that there is an eigenvalue that exceeds a certain bound without stipulating an exact value. In the following, we refer to these cases as functional motifs of the first and second kinds, respectively.

**Fig. 1.**
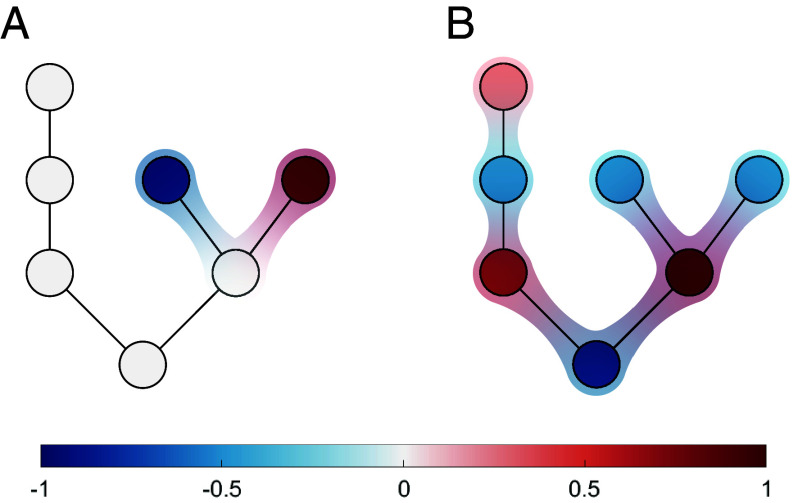
Comparison between localized and delocalized eigenvectors of a Jacobian matrix. The example shows two different eigenvectors of the Jacobian matrix from the same system as heatmaps. Nodes corresponding to zero elements in the eigenvector (white) have no impact on the associated eigenvalue. The exploitative competition motif localizes an eigenvector (*A*), such that the corresponding eigenvalue becomes independent of nodes outside the motif. By contrast, typical eigenvectors (*B*), which also exist in the same system, are delocalized and hence depend on all of the nodes. This illustrates that eigenvector localization, if it occurs in a system, can result in the formation of functional motifs.

We start by considering motifs of the first kind, where the presence of a motif directly causes the presence of a specific eigenvalue. Generally, the eigenvalues of a large matrix do not originate from a small motif but emerge from the matrix as a whole (Fig. 1). To explore this more deeply, we build on results from matrix perturbation theory (19), which show that the set of nodes that cause an eigenvalue is indicated by the corresponding eigenvector. Eigenvector plays a two-fold role: First, it indicates a direction in which a system can escape if the corresponding eigenvalue is positive (18). Second, and more importantly, the eigenvector indicates the nodes to which the eigenvalue is sensitive (19).

In a system of N variables, Jacobian eigenvectors contain N elements, each corresponding to one of the variables. If a normalized eigenvector has a large element, then the corresponding variable has a strong impact on the associated eigenvalue. By contrast, a small element means that the corresponding variable has only a weak impact and a zero element means that an eigenvalue is (at least locally) independent of the variable. We can thus say that a network has a functional stability motif of the first kind if there is a positive eigenvalue for which the corresponding eigenvector is zero in all nodes outside the motif.

## Exploitative Competition Example

Common derivations of the competitive exclusion principle typically start by considering a system of two populations of specialist competitors of the form

[2a] $$\begin{array}{cc} \dot{X} & =G_{x}\left(R\right)X-m_{x}X \end{array}$$

[2b] $$\begin{array}{cc} \dot{Y} & =G_{y}\left(R\right)Y-m_{y}Y, \end{array}$$

where X and Y are abundances or biomasses of the two populations, m are the respective mortality rates, G are the growth rates, and the dot over a variable denotes the change in time. The variable R is the resource that both of the competing consumer populations are exploiting and which possibly interacts with many other species that do not have a direct impact on the competing consumers. The dynamics of R and any other variables in the system are not essential to the argument that is made and are hence omitted here.

One can show that the coexistence of the species is only feasible if one of the parameters (e.g., $m_{x}$) is chosen exactly right (10, 20), which is thought to be implausible in nature. However, this reasoning has been criticized as it applies a genericity argument to a system where the degeneracy is built into the modeling assumptions (5).

Let us instead consider the stability of the steady state if one exists. For any such steady state the eigenvector equation is solved by

[3] $$\begin{array}{c} \underset{J}{\underbrace{\left[\begin{array}{ccccc} 0 & 0 & a_{1} & 0 & \cdots \\ 0 & 0 & a_{2} & 0 & \cdots \\ b_{1} & b_{2} & c_{11} & c_{12} & \ldots \\ 0 & 0 & c_{21} & c_{22} & \ldots \\ \vdots & \vdots & \vdots & \vdots & \ddots \end{array}\right]}}\underset{v}{\underbrace{\left(\begin{array}{c} b_{2}\\ -b_{1}\\ 0\\ 0\\ \vdots \end{array}\right)}}=\underset{\lambda v}{\underbrace{\left(\begin{array}{c} 0\\ 0\\ 0\\ 0\\ \vdots \end{array}\right)}}, \end{array}$$

where the first row and column of the matrix correspond to the X, the second to Y, and the third to R. The Jacobian matrix has a block of zeros (blue) in the top-left as self-interactions vanish as a consequence of the linearity of gain and loss rates with respect to the consumer populations (21), and the two competitors only interact with each other and the rest of the network via their resource R (third row). The only nonzero elements that we find in the first two columns of the matrix are hence $b_{1}$ and $b_{2}$, which describe the consumers’ impact on the resource and whose values will depend on the specific system under consideration. However, a vector of the form v shown in the equation will always be an eigenvector of the matrix with a corresponding eigenvalue λ=0.

The example shows that the exploitative competition motif implies the presence of a zero eigenvalue, regardless of the interactions in the rest of the system (represented by $c_{\mathrm{ij}}$). Hence, any system involving competition between uncontrolled consumers described by Eq. **2b** precludes asymptotic stability of the network (see *SI Appendix* for details). We can therefore say that the two species whose dynamics are described by Eq. **2b** form a functional stability motif, as their joint presence in the absence of internal regulation inherently disrupts stability.

Note that the infeasibility of stationary coexistence is tied to the presence of this zero eigenvalue through the implicit function theorem of calculus, which allows us to compute how stationary states change as parameter values shift. If the Jacobian has a zero eigenvalue, the solutions diverge, signaling an abrupt change in the solution and typically annihilation of the equilibrium.

## Functional Stability Motifs Are Rare

The exploitative competition example illustrates that an eigenvalue is determined by a small motif if the eigenvector elements that correspond to nodes outside the motif are zero. Such eigenvectors that are zero except in a small motif are called localized eigenvectors. We can now state that a functional stability motif of the first kind requires the presence of a localized eigenvector of the Jacobian.

In connected networks exact localization, where eigenvector elements outside a motif become exactly zero, appears only in response to the presence of certain symmetries in the network (22). In unweighted networks, these symmetries and hence localized eigenvectors are common (23). However, in network representations of Jacobians, which are weighted networks, link weights must also satisfy certain symmetry conditions to allow for eigenvector localization. Specifically, a vector is a localized eigenvector for a specific motif if: *i*) it is an eigenvector for the motif in isolation, and *ii*) the eigenvector elements are zero on those nodes in the motif to which outside nodes are allowed to attach.

For example, consider the modified system

[4a] $$\begin{array}{cc} \dot{X} & =F\left(R\right)X-mX^{1+p} \end{array}$$

[4b] $$\begin{array}{cc} \dot{Y} & =F\left(R\right)Y-mY^{1+p} \end{array}$$

which has a stationary state at $X^{*}=Y^{*}>0$. The Jacobian matrix for the motif in isolation, including R, has the structure

[5] $$\begin{array}{c} J=\left[\begin{array}{ccc} d & 0 & a\\ 0 & d & a\\ b & b & c \end{array}\right], \end{array}$$

where a,b,c,d depend on the specifics of the system. For this matrix, the vector ${\left(1,-1,0\right)}^{\prime }$ is an eigenvector and the element corresponding to R is zero, creating a point where other parts can be attached. Hence, this motif is a functional motif of the first kind.

The eigenvalue corresponding to the localized vector is $\lambda =d=-mp{\left(X^{⋆}\right)}^{p}$. Hence, its presence in the network destabilizes the system if p≤0, but not if p>0, which is a known result (5).

In this example the localization appears because we assumed the two competitors behave identically, preserving complete symmetry between the species (24). While it is possible to find nonsymmetric examples where fortuitous cancellations happen to meet the conditions, these are likewise special cases. When searching for a vector that meets the two conditions, the condition *i*) alone reduces the choice down to a narrow set of candidates, as the motif in isolation only has a finite set of eigenvectors. The probability that any of these eigenvectors is able to meet condition *ii*) exactly is of measure zero, except if a symmetry exists. The exploitative competition motif with linear rates escapes the logic above because the motif block contains only zeroes, because every vector is an eigenvector of a zero matrix, which makes it easy to meet the first condition.

## Routes to Functional Stability Motifs

There are some additional ways in which functional stability motifs of the first kind can arise. While the implications for ecology are mostly well-known, we find it valuable to provide a brief but comprehensive overview. We have seen that the original exploitative competition motif is a functional motif because it assumes linear rates and absence of internal interactions within the motif. This will create a functional motif whenever we have sufficient degrees of freedom left to satisfy the condition on adjacent nodes, i.e., whenever the number of nodes within the motif is greater than the number of adjacent nodes, which is equivalent to the general formulation of the competitive exclusion principle.

Our second example was a functional motif due to the assumption of identical dynamics. A motif is a functional motif if there is a nontrivial symmetry in the Jacobian matrix (this is the case whenever the order of variables can be changed while leaving the matrix unchanged, see *SI Appendix*). In practice this will require the assumption that there are sets of variables that behave identically. In ecology, this assumption could be warranted as an approximation as there are many examples of species that appear to be functionally redundant.

A trivial way to achieve eigenvector localization, mentioned for completeness, is disconnection or unidirectional connection. If a part of a system interacts with the rest of the system not at all or only unidirectionally then also eigenvalues will remain localized in their respective parts. Such an isolation of a part of the system is rare and hence this scenario will be irrelevant in most applications.

If the conditions for exact localization of eigenvectors are not met, we can sometimes still have approximate localization reminiscent of Anderson localization in physical systems (25). In this case, eigenvectors from the motif spill into the rest of the network, but the eigenvector elements decay exponentially with increasing distance from the motif, leading to an eigenvalue that is nearly independent of the nodes outside the motif.

We expect approximate localization when a subgraph can sustain a very different eigenvalue from the rest of the network. For example, approximately localized eigenvectors arise if a tightly knit community is only weakly interacting with a wider network that is sparser or operating on different time scales. Approximate localization may thus provide a post factum justification for modeling, say, an aquatic food web within a lake separately from the surrounding terrestrial system (26), or modeling a mammalian food web without considering invertebrates and microbes (27). In these cases, we would expect eigenvectors to become approximately localized, making the system insensitive to the wider world. Hence, for example, the lake food web can become a functional motif in the broader ecosystem.

In summary, this section shows that there are a number of scenarios that lead to functional motifs of the first kind. However, these scenarios are relatively rare and have consequences that are already well known in ecology. Let us therefore now turn to functional motifs of the second kind, which guarantee that one eigenvalue will have at least a certain value.

In general dynamical systems, the presence of a certain subgraph does not constrain the Jacobian eigenvalues of the system as a whole. Therefore, functional stability motifs of the second type only exist under specific circumstances but are ubiquitous in the subclass of dynamical systems that have symmetric Jacobian matrices, meaning $J_{\mathrm{ij}}=J_{\mathrm{ji}}$ for all i,j. Such systems arise for example in the modeling of coupled oscillators, meta-populations, and epidemics on networks.

For a symmetric matrix, application of Cauchy’s interlacing theorem (28) proves that the largest eigenvalue of the entire system must be at least as large as the largest eigenvalue of any subgraph that we find in the system. Hence, in systems with symmetric Jacobians, any subgraph can act as a functional stability motif.

## Functional Reactivity Motifs

Besides local asymptotic stability, many other notions of stability have been proposed in ecology and beyond. For example, persistence and permanence quantify the ability of populations to survive in the system, which is of more direct relevance to environmental question, but harder to link to motifs. An analysis by Stouffer and Bascompte found no direct relationship, though they note that motifs that had a higher persistence in isolation are also more common in food webs (16).

**Fig. 2.**
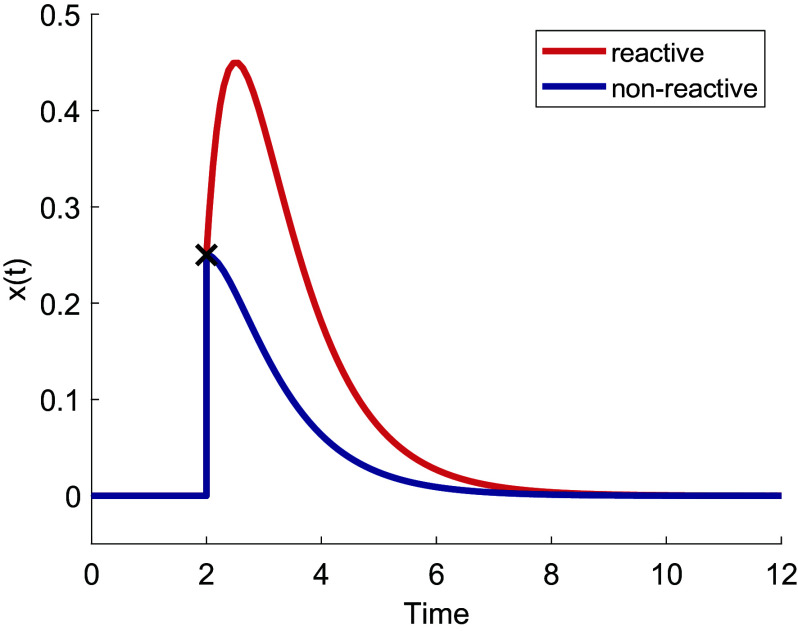
Behavior of reactive and nonreactive stable systems. A system in stable equilibrium responds to a sufficiently small perturbation (cross) by eventually returning to its equilibrium state (here, 0). In nonreactive systems, this return is uniform (blue). Conversely, in a reactive system, a transient amplification of the perturbation occurs (red).

Here we focus on reactivity (17, 29–31), which describes the system’s initial amplification of a perturbation (Fig. 2). Reactivity is defined as the initial growth rate of a worst-case perturbation, which can be computed as the leading eigenvalue of the symmetric part of the Jacobian $S=(J+J^{\prime })/2$ (17). If the reactivity is positive then perturbations are initially amplified by the system, and the system is said to be reactive. If the reactivity is negative then perturbations are attenuated and the system is said to be nonreactive. This means that any unstable system is reactive, while stable systems can be reactive or nonreactive.

Because reactivity is computed as an eigenvalue of a symmetric matrix S, the interlacing theorem implies that the largest eigenvalue of S must be equal or greater than the largest eigenvalue of any motif found in S. In other words, observing a certain amount of reactivity in a part of the network provides lower bound for the reactivity of the system. Thus every subgraph of a network is a functional reactivity motif.

The interlacing theorem shows that reactivity that is generated in a part of the network cannot be compensated by structures outside the part. This is exciting because it opens up the possibility to search for parts of ecological networks that are major sources of reactivity and thus pose a systemic risk when disturbed.

The interlacing theorem also tells us that the reactivity of the full system is greater than the reactivity of any isolated subsystem or part. Hence, we need to ask whether reactivity is predominantly a systemic property such that its major share emerges only when nearly the entire network is considered. If this were the case, analyzing the reactivity found in small subgraphs would be practically useless. Conversely, if a proportion of the reactivity is rooted in small motifs, searching for these localized sources of reactivity in real-world systems becomes a worthwhile endeavor.

To explore the scale at which reactivity emerges, we generated plausible food web topologies with 15 species using the niche model (32). We then converted the food web topologies into dynamical models using the generalized modeling approach (21), with parameters that are drawn randomly from plausible parameter ranges (*SI Appendix*). From the resulting Jacobian matrices we rejected those that corresponded to unstable and nonreactive stable states, as we are interested in systems where reactivity is a relevant concern. We thus created two sets containing $10^{4}$ Jacobians for stable but reactive systems. One set contained random topologies with randomly chosen parameters (set 1), whereas the second set contained Jacobians with randomly chosen parameters for a single fixed network topology (set 2).

**Fig. 3.**
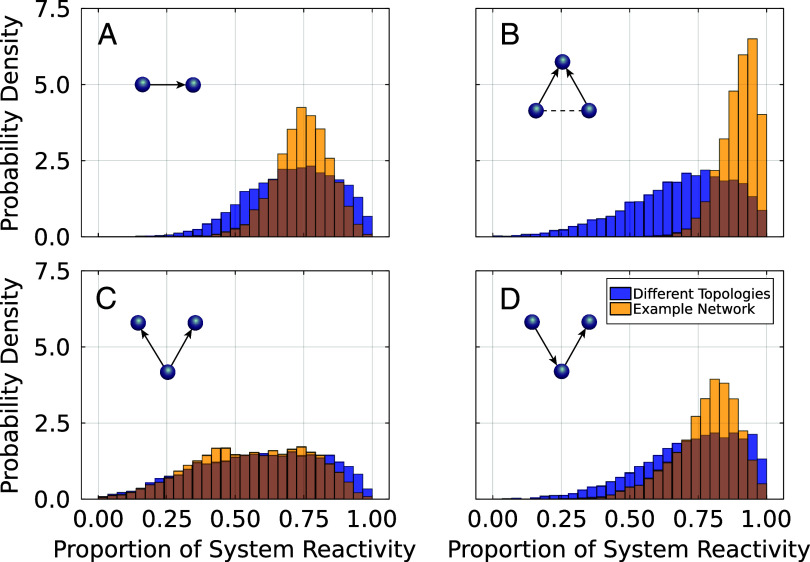
Contribution of individual motif to system reactivity. The histograms show the proportion of total system reactivity contained in the network’s most reactive instances of the particular motifs indicated in the upper left corner of each subplot, across $10^{4}$ realizations. Results are shown for an ensemble of networks with different topologies (blue) and for a specific example network (orange, see also Fig. 4 for the network structure), and predator–prey (*A*), apparent competition (*B*), exploitative competition (*C*), and tritrophic chain motifs (*D*). This shows that reactivity frequently stems from distinct, highly localized sources within a larger network.

To assess whether small motifs can explain a significant proportion of system-wide reactivity, we consider individual predator–prey interactions as well as small subgraphs, including all ecologically sensible subgraphs of size three. We examined the most reactive instance of each motif and compared its reactivity to that of the entire system (Fig. 3).

The analysis reveals broad variation in reactivity across motifs, with some motifs containing only small or moderate amounts while others cause almost as much reactivity as the entire system. In the most extreme case, we find that the most reactive predator–prey link alone causes ≥99.9% of the total network reactivity in set 1 (≥99.3% in set 2). When we consider three-node motifs, this number rises to ≥99.9% for both sets (see *SI Appendix* for discussion of larger subgraphs). Furthermore, in 88.3% of webs (set 1) or 98.2% (set 2) the most reactive link alone contributes at least half of the system’s reactivity. If we consider subgraphs of size three this value increases to 98.7% (set 1) and 99.9% (set 2). These results show that, typically, the major portion of reactivity is generated in small localized parts of the network, suggesting that it may be useful to look for such isolated sources of reactivity in nature.

**Fig. 4.**
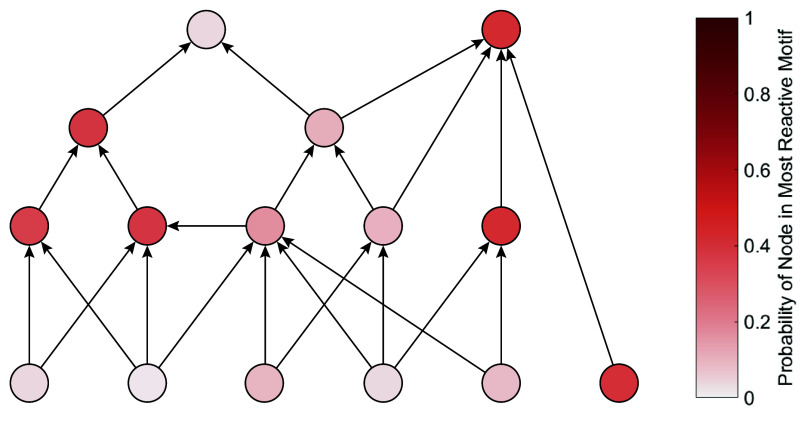
Node participation in the most reactive apparent competition motif. Shown is the example food web topology. The color of the nodes indicates the probability that a node participates in the most reactive apparent competition motif. Darker shades of red correspond to higher probabilities. The figure shows that the most reactive apparent competition motif most likely consists of the three nodes on the right or the three nodes on the left of the figure. This illustrates that specific network structures drive network reactivity.

To facilitate the search for reactive motifs in nature it is useful to quantify the extent to which a motif’s reactivity is rooted in its topological structure versus the nonlinearity of its processes. We therefore examine set 2 where the topology of the network remains fixed, but the dynamical parameters are randomized. In this network apparent competition (AC) motifs are the main source of reactivity, with the most reactive AC motif causing more than 90% of the system’s reactivity in over half the networks. Plotting the participation of the nodes in the most reactive motif (Fig. 4) reveals that two specific apparent competition motifs were the most reactive in 73% of the realizations. These results show that topology plays a significant role in the reactivity of motifs, though dynamical factors can still determine which motif is ultimately the most reactive.

Because the reactivity of a small motif is generated entirely within the motif, a detailed analysis can be conducted to pinpoint factors contributing to reactivity. While a detailed mathematical discussion is beyond the scope of the present paper we conjecture that long predator–prey links, bridging multiple trophic levels contribute strongly to reactivity. Our results from the analysis of niche webs (Fig. 3) show that apparent competition and tritrophic chain motifs have the highest likelihood of explaining a high proportion of the reactivity in the system, whereas the exploitative competition motif seems to have a lesser effect.

## Conclusions

We examined the conditions under which small parts of a network act as functional motifs, such that their presence has systemic implications that cannot be undone by the wider network. We presented an alternative approach to the role of the exploitative competition motif in the competitive exclusion principle and used it to explain why similar functional stability motifs are unlikely. We then provided an overview of ways in which small subgraphs can gain functional importance, which highlighted reactivity as an ecological concept for which every subgraph acts as a functional motif.

Our numerical explorations showed that motifs with two or three species can typically explain a significant proportion of the reactivity of model networks. It is therefore plausible that real-world food webs contain such motifs that are strong, localized drivers of reactivity. Hence, efforts should be made to identify these motifs in natural webs, as they could act as strong amplifiers of disturbances.

The search for reactivity motifs in the real world can benefit from mathematical analysis of small motifs, which can highlight the specific topologies and nonlinearities that cause high values of reactivity to appear. To corroborate the existence of these reactivity motifs in nature will thus require both data analysis from real-world food webs and further mathematical work.

While we have used food webs as a primary example, we believe that the results apply to a wide class of dynamical systems that are at risk from perturbations, ranging from power grids and supply chains to networks of social influence. Due to the mathematical properties of reactivity presented here, we can say that in these networks, every subgraph will be a reactivity motif. However, determining how much of a system’s reactivity is rooted in small subgraphs and how much arises only at the systemic level in these applications will require subsequent studies.

We hope future research, perhaps inspired by this work, will deepen our understanding of which system properties arise from parts of a network and which emerge from the network as a whole. In the former case, functional motifs can be identified to narrow the focus to the most critical parts. In the latter case, details should not matter, enabling the use of coarse-grained models. Hence, in both cases, a simplification can be achieved. Further insights into when to pursue one route or the other might ultimately lead to a general theory for the mathematical modeling of complex systems.

## Materials and Methods

### Reactivity.

Reactivity measures the maximum relative growth rate of the deviation from a steady state after a sufficiently small perturbation δ. Following Neubert and Caswell (17), we write it as

[6] $$\begin{array}{c} r=\max\frac{1}{|\delta |}\frac{\text{d}}{\text{d}t}|\delta |, \end{array}$$

where

[7] $$\begin{array}{c} |\delta |=\sqrt{\delta^{\prime }\delta } \end{array}$$

is the magnitude of the deviation δ away from the steady state.

If the growth rate is negative, the perturbation decays, and the system returns to its steady state. Steady states where this is the case are said to be nonreactive. If the growth rate is positive, the perturbation is amplified, causing the system to move initially further away from its equilibrium. Steady states where this is the case are called reactive.

Neubert and Caswell (17) showed that r can be computed as the leading eigenvalue of the symmetric part of the Jacobian J

[8] $$\begin{array}{c} S=\frac{J+J^{\prime }}{2}\;. \end{array}$$

### Interlacing Theorem.

Consider a symmetric matrix and a principal submatrix that is created by removing one row and column from the matrix. Use $\lambda_{n}$ to denote the eigenvalues of the matrix and $\kappa_{n}$ to denote the eigenvalues of the submatrix, both numbered in descending order. Cauchy’s interlacing theorem (28) then implies that the eigenvalues must obey the condition

[9] $$\begin{array}{c} \lambda_{N}\leq \kappa_{N-1}\leq \lambda_{N-1}\leq \ldots \leq \kappa_{2}\leq \lambda_{2}\leq \kappa_{1}\leq \lambda_{1}. \end{array}$$

This implies that the leading eigenvalue of the entire system $\lambda_{1}$ must always be greater or equal to the leading eigenvalue of any part of the system.

### Generalized Food Web Model.

Generalized models do not restrict the processes in the model to specific functional forms (21, 33), which means that a generalized model is able to accommodate uncertainty over the precise functional form, and a single generalized model can represent a whole class of similar ecological models. This uncertainty can then be propagated through the computation of the Jacobian matrix computation, where it is ultimately captured in a set of ecologically interpretable parameters. Generalized models thus have almost the same interpretability as conventional models, while rivaling the efficiency of random matrix models.

## Supplementary Material

Appendix 01 (PDF)

## Data Availability

Code data have been deposited in https://github.com/MelHabm/FunctionalMotifsInFoodwebs (34) (DOI: 10.5281/zenodo.17038707) (35).

## References

[1] G. F. Gause, The Struggle for Existence (Courier Dover Publications, 1934).

[2] G. Hardin, The competitive exclusion principle: An idea that took a century to be born has implications in ecology, economics, and genetics. Science **131**, 1292–1297 (1960). 14399717 10.1126/science.131.3409.1292

[3] S. A. Levin, Community equilibria and stability, and an extension of the competitive exclusion principle. Am. Nat. **104**, 413–423 (1970).

[4] R. H. MacArthur, Population ecology of some warblers of northeastern coniferous forests. Ecology **39**, 599–619 (1958).

[5] T. Gross, A. M. Edwards, U. Feudel, The invisible niche: Weakly density-dependent mortality and the coexistence of species. J. Theor. Biol. **258**, 148–155 (2009). 19490872 10.1016/j.jtbi.2009.01.018

[6] M. A. McPeek, Intraspecific density dependence and a guild of consumers coexisting on one resource. Ecology **93**, 2728–2735 (2012). 23431602 10.1890/12-0797.1

[7] R. A. Armstrong, R. McGehee, Competitive exclusion. Am. Nat. **115**, 151–170 (1980).

[8] P. L. Chesson, “Interactions between environment and competition: How fluctuations mediate coexistence and competitive exclusion” in Community Ecology: A Workshop held at Davis, CA, April 1986, A. Hastings, Ed. (Springer, 1988), pp. 51–71.

[9] A. M. Siepielski, M. A. McPeek, On the evidence for species coexistence: A critique of the coexistence program. Ecology **91**, 3153–3164 (2010). 21141177 10.1890/10-0154.1

[10] D. Tilman, Resource Competition and Community Structure (Princeton University Press, 1982), vol. 296. 7162524

[11] P. A. Abrams, The effect of density-independent mortality on the coexistence of exploitative competitors for renewing resources. Am. Nat. **158**, 459–470 (2001). 18707301 10.1086/323113

[12] J. J. Borrelli, Selection against instability: Stable subgraphs are most frequent in empirical food webs. Oikos **124**, 1583–1588 (2015).

[13] A. R. Cirtwill, K. L. Wootton, Stable motifs delay species loss in simulated food webs. Oikos **2022**, e09436 (2022).

[14] K. E. Anderson, A. K. Fahimipour, Body size dependent dispersal influences stability in heterogeneous metacommunities. Sci. Rep. **11**, 17410 (2021). 34465802 10.1038/s41598-021-96629-5 PMC8408130

[15] P. Lawton, A. K. Fahimipour, K. E. Anderson, Interspecific dispersal constraints suppress pattern formation in metacommunities. Philos. Trans. B **379**, 20230136 (2024). 10.1098/rstb.2023.0136 PMC11391288 38913053

[16] D. B. Stouffer, J. Bascompte, Understanding food-web persistence from local to global scales. Ecol. Lett. **13**, 154–161 (2010). 19968697 10.1111/j.1461-0248.2009.01407.x

[17] M. G. Neubert, H. Caswell, Alternatives to resilience for measuring the responses of ecological systems to perturbations. Ecology **78**, 653–665 (1997).

[18] J. Guckenheimer, P. Holmes, Nonlinear Oscillations, Dynamical Systems, and Bifurcations of Vector Fields (Springer, New York, ed. 3, 1990).

[19] G. W. Stewart, J. G. Sun, Matrix Perturbation Theory (Academic Press Inc, Cambridge, MA, 1990).

[20] P. Abrams, The theory of limiting similarity. Annu. Rev. Ecol. Syst. **14**, 359–376 (1983).

[21] T. Gross, U. Feudel, Generalized models as a universal approach to the analysis of nonlinear dynamical systems. Phys. Rev. E **73**, 016205 (2006). 10.1103/PhysRevE.73.016205 16486256

[22] B. D. MacArthur, R. J. Sánchez-García, Spectral characteristics of network redundancy. Phys. Rev. E Stat. Nonlinear Soft Matter Phys. **80**, 026117 (2009). 10.1103/PhysRevE.80.026117 19792210

[23] A. Nyberg, T. Gross, K. E. Bassler, Mesoscopic structures and the Laplacian spectra of random geometric graphs. J. Complex Netw. **3**, 543–551 (2015).

[24] H. Aufderheide, “Implications of eigenvector localization for dynamics on complex networks,” PhD thesis, TU Dresden (2014).

[25] P. W. Anderson, Absence of diffusion in certain random lattices. Phys. Rev. **109**, 1492 (1958).

[26] A. K. Fahimipour, A. M. Hein, The dynamics of assembling food webs. Ecol. Lett. **17**, 606–613 (2014). 24589244 10.1111/ele.12264

[27] J. D. Yeakel , Collapse of an ecological network in ancient Egypt. Proc. Natl. Acad. Sci. U.S.A. **111**, 14472–14477 (2014). 25201967 10.1073/pnas.1408471111 PMC4210013

[28] S. G. Hwang, Cauchy’s interlace theorem for eigenvalues of Hermitian matrices. Am. Math. Mon. **111**, 157–159 (2004).

[29] R. E. Snyder, What makes ecological systems reactive? Theor. Popul. Biol. **77**, 243–249 (2010). 20346368 10.1016/j.tpb.2010.03.004

[30] A. Hastings , Effects of stochasticity on the length and behaviour of ecological transients. J. R. Soc. Interface **18**, 20210257 (2021). 34229460 10.1098/rsif.2021.0257 PMC8261213

[31] Y. Yang, K. Z. Coyte, K. R. Foster, A. Li, Reactivity of complex communities can be more important than stability. Nat. Commun. **14**, 7204 (2023). 37938574 10.1038/s41467-023-42580-0 PMC10632443

[32] R. J. Williams, N. D. Martinez, Simple rules yield complex food webs. Nature **404**, 180–183 (2000). 10724169 10.1038/35004572

[33] J. C. Massing, T. Gross, Generalized structural kinetic modeling: A survey and guide. Front. Mol. Biosci. **9**, 825052 (2022). 35573734 10.3389/fmolb.2022.825052 PMC9098827

[34] M. Habermann, T. Gross, FunctionalMotifsInFoodwebs. GitHub. https://github.com/MelHabm/FunctionalMotifsInFoodwebs/tree/v1.0.0. Accessed 2 September 2025.

[35] M. Habermann, T. Gross, FunctionalMotifsInFoodwebs (v1.0.0). Zenodo. 10.5281/zenodo.17038707. Deposited 2 September 2025.

