# Supplementary material for: Functional motifs in food webs and networks
Source: Proc Natl Acad Sci U S A. 2026 Jan 29;123(5):e2521927123. doi: 10.1073/pnas.2521927123 (PMC12867680; doi:10.1073/pnas.2521927123)
Supplement: Supplementary file 1 — Appendix 01 (PDF) [file pnas.2521927123.sapp.pdf]

Supporting Information for

Functional Motifs in Foodwebs and Networks

Melanie Habermann, Ashkaan K. Fahimipour, Justin D. Yeakel and Thilo Gross

Correspondence to: mel2.habermann@gmail.com

This PDF file includes:

Supporting text
Fig. S1
SI References

Supporting Information Text

Generalized Foodweb Model. The model is based on the work of Thilo Gross and Ulrike Feudel (1).

Consider a system with N different populations of species X_1, $X_2 \ldots$, X_N. We can describe the dynamics of each population by an ODE of the form

$$\dot{X}_i = S_i(X_i) + \eta_i F_i(\boldsymbol{X}) - M_i(X_i) - \sum_{j=1}^{N} L_{ji}(\boldsymbol{X}) \,, \qquad [1]$$

where X_i represents the abundance of the species in question and $S_i(X_i)$, $F_i(\boldsymbol{X})$, $M_i(X_i)$, and $L_{ji}(X_i)$ are non-linear functions for the population gains and losses. Depending on the species, population growth is either due to primary production $S(X)$ or predation on other species $F(X)$. Since the consumed food is not entirely converted to a population gain for the predator, we include a conversion factor η. Population losses can either be caused by natural mortality (e.g., disease, age) or predation by a different species j. We do not consider cannibalistic behaviour in this study.

A predator-prey relationship necessitates a link between the two functions $F_i(\boldsymbol{X})$ and $L_{ji}(\boldsymbol{X})$. Additionally, it should account for the option that a predator can consume multiple prey species. To represent this more realistically, we introduce two auxiliary variables, $T_j(\boldsymbol{X})$ and $C_{ji}(X_j)$. $T(X)$ determines the overall food supply for species j whereas $C_{ji}(X_j)$ defines the contribution of species i in the diet of j. We can define

$$T_j(\boldsymbol{X}) = \sum_{i=1}^{N} C_{ji}(X_i) \,. \qquad [2]$$

By employing $T_j(\boldsymbol{X})$ we can redefine $F_j(\boldsymbol{X})$ as $F_j(T_j(\boldsymbol{X}), X_j)$.

Now, when we factor in the portion of the total food supply that species i contributes to the predator's diet, we can modify the predation loss function as follows:

$$L_{ji}(\boldsymbol{X}) = \frac{C_{ji}(X_j)}{T_j(\boldsymbol{X})} F_j(T_j(\boldsymbol{X}), \boldsymbol{X_j}) \,. \qquad [3]$$

Normalizing the Model. For a model with the complexity of a foodweb we can be sure of at least one steady state $\boldsymbol{X}*$, where each state variable has its equilibrium value X_i^* (1, 2). To simplify things, we use this to define normalized state and auxiliary variables as follows

$$x_i = \frac{X_i}{X_i^*} \,, \qquad [4]$$

$$t_j(\boldsymbol{x}) = \frac{T_j(\boldsymbol{X})}{T_j^*} \,, \qquad [5]$$

$$c_{ji}(x_i) = \frac{C_{ji}(X_i)}{C_{ji}^*} \,. \qquad [6]$$

We also introduce normalized versions of each process function $P_i(X_i)$ involved in the model:

$$p_i(x_i) = \frac{P_i(X_i)}{P_i(X_i^*)} \,, \qquad [7]$$

where $P_i(X_i^*)$ is the process function in the steady state. For better readability in the following parts, we use P_i^* instead of $P_i(X_i^*)$.

Rewriting the model from Eq. 1 with these normalized quantities yields

$$\dot{x}_i = \frac{S_i^*}{X_i^*} s_i(x_i) + \frac{F_i^*}{X_i^*} f_i(\boldsymbol{x}, x_i) - \frac{M_i^*}{X_i^*} m_i(x_i) - \sum_{j=1}^{N} \frac{L_{ji}^*}{X_i^*} l_{ji}(\boldsymbol{x}) \,, \qquad [8]$$

where

$$l_{ji}(\boldsymbol{x}) = \frac{C_{ji}^* F_j^*}{T_j^* L_{ji}^*} \frac{c_{ji}}{t_j} f_j(t_j(\boldsymbol{x}, x_j) = \frac{c_{ji}}{t_j} f_j(t_j(\boldsymbol{x}, x_j) \,. \qquad [9]$$

For the total food supply $t_j(\boldsymbol{x})$, we get

$$t_j(\boldsymbol{x}) = \sum_{i=1}^{N} \frac{Cji^*}{T_j^*} c_{ji}(x_i) \,. \qquad [10]$$

Scale Parameters. The model can be further simplified by introducing a set of easily interpretable. These scale parameters allow the quantification of biomass flow. In its simplest form we can describe the flow for a whole population. It is also important to note that gains and losses that produce this flow are often the sum of multiple processes. Using scale parameters, we can specify the proportions of how these processes contribute to the gains and losses (2).

If we consider the system in its steady state, $\dot{\boldsymbol{x}}$ is by definition zero. Because we normalized the model in the previous steps, all state variables and process rates are now one. If we take this into consideration, we can write the model in its steady state as

$$0 = \frac{S_i^*}{X_i^*} + \frac{F_i^*}{X_i^*} - \frac{M_i^*}{X_i^*} - \sum_{j=1}^{N} \frac{L_{ji}^*}{X_i^*} \, . \tag{11}$$

We are left with a sum of constant values that we use as our scale parameters.

For the overall biomass flow of species i, we define a parameter

$$\alpha_{xi} = \frac{S_i^*}{X_i^*} + \frac{F_i^*}{X_i^*} - \frac{M_i^*}{X_i^*} - \sum_{j=1}^{N} \frac{L_{ij}^*}{X_i^*} \, . \tag{12}$$

This will leave us with a number of N parameters just for the overall biomass flow of all N species. We can reduce this number by one if we rescale the time for all species with the turnover rate of the species on the lowest trophic level α_1.

The two gain terms for primary production and predation account for the positive biomass flow. To quantify the relative contributions of each process we use

$$\rho_{xi} = \frac{1}{\alpha_{xi}} \frac{S_i^*}{X_i^*} \, , \tag{13}$$

$$\hat{\rho}_{xi} = 1 - \rho_{xi} = \frac{1}{\alpha_{xi}} \frac{F_i^*}{X_i^*} \, . \tag{14}$$

We also define parameters for the relative contribution to the biomass loss

$$\sigma_{xi} = \frac{1}{\alpha_{xi}} \frac{M_i^*}{X_i^*} \, , \tag{15}$$

$$\hat{\sigma}_{xi} = \frac{1}{\alpha_{xi}} \sum_{j=1}^{N} \frac{L_{ij}^*}{X_i^*} \tag{16}$$

For species that are hunted by multiple predators, the proportional loss caused by each is represented by additional branching parameters

$$\beta_{ji} = \frac{1}{\alpha_{xi}\hat{\sigma}_{xi}} \frac{L_{ij}^*}{X_i^*} \, . \tag{17}$$

In Eq. 10 we already saw that each prey species contributes only a fraction

$$\frac{C_{ji}^*}{T_j^*} \tag{18}$$

to a predator's diet. Since these are also constant values, we redefine them as additional scale parameters

$$\chi_{ji} = \frac{C_{ji}^*}{T_j^*} \, , \tag{19}$$

With this Eq. 10 becomes

$$t_j(\boldsymbol{x}) = \sum_{i=1}^{N} \chi_{ji} c_{ji}(x_i) \, . \tag{20}$$

Including the set of scale parameters in Eq. 11 leaves us with the functions

$$\dot{x}_i = \alpha_i \left(\rho_{xi} s_i(x_i) + \hat{\rho}_{xi} f_i(\boldsymbol{x}, x_i) - \sigma_{xi} m_i(x_i) - \hat{\sigma}_{xi} \sum_{j=1}^{N} \beta_{ji} l_{ji}(\boldsymbol{x}) \right) \, . \tag{21}$$

where

$$l_{ji}(\boldsymbol{x}) = \frac{c_{ji}}{t_j} f_j(t_j(\boldsymbol{x}, x_j) \, , \tag{22}$$

$$t_j(\boldsymbol{x}) = \sum_{i=1}^{N} \chi_{ji} c_{ji}(x_i) \, . \tag{23}$$

To reduce the number of parameters in the following calculations we assume that predation is the main cause of death in prey species and affects the species to a significantly larger extent, so that $m_i(x_i)$ can be neglected in these cases. For this, we set $\sigma_{xi} = 0$ and $\hat{\sigma}_{xi} = 1$ for all prey populations. Only for top predators, we assume the opposite to be true.

We can also safely assume that primary production is a trait we will only find on the lowest trophic level. To represent this in our model we set $\rho_{xi} = 1$ and $\hat{\rho}_{xi} = 0$ for primary producers and vice versa for every species on a higher trophic level.

Computation of the Jacobian and Corresponding Symmetric Matrix. To compute the Hermitian matrix we first have to construct the system's Jacobian. We introduce another set of parameters (i.e., exponent parameters) that will represent the partial derivatives of our general process functions. Again, you can find sensible interpretations for these parameters. In general, we can understand them as the non-linearity of the corresponding processes.

The new set of parameters includes

$$s_{xi} := \left.\frac{\partial s(x_i)}{\partial x_i}\right|_*, \qquad f_{xi} := \left.\frac{\partial f(t_i(\boldsymbol{x}), x_i)}{\partial x_i}\right|_*, \qquad f_{tj} := \left.\frac{\partial f(t_j(\boldsymbol{x}), x_j)}{\partial t_j}\right|_*,$$

$$\lambda_{ji} := \left.\frac{\partial c_{ji}}{\partial x_i}\right|_*, \qquad \text{and } m_{xi} := \left.\frac{\partial m(x_i)}{\partial x_i}\right|_*. \qquad [24]$$

Now that we have defined the exponent parameters, we can use them to construct our Jacobian matrix $\mathbf{J}$. The resulting Jacobian has the diagonal entries

$$\mathbf{J}_{ii} = \alpha_i \left(\rho_{xi} s_{xi} + \hat{\rho}_{xi} f_{xi} - \sigma_{xi} m_{xi} - \hat{\sigma}_{xi} \sum_{j=1}^{N} \beta_{ji} \lambda_{ji} (\chi_{ji}(f_{tj} - 1) + 1) \right) \qquad [25]$$

and non-diagonals

$$\mathbf{J}_{ni} = \alpha_n \left(\hat{\rho}_{xn} \lambda_{ni} \chi_{ni} f_{tn} - \hat{\sigma}_{xn} \sum_{j=1}^{N} \beta_{jn} \lambda_{ji} \chi_{ji} (f_{tj} - 1) \right). \qquad [26]$$

Following the step outlined in the main text in Eq. 15 we can calculate the Hermitian matrix. It is important to note that the diagonal entries of $\mathbf{S}$ are identical to those in the Jacobian. These diagonal elements indicate the effect that a species has on itself, and they provide insights into whether the network contains reactive nodes or not. If we can already identify reactive nodes (i.e., positive values on the main diagonal), we can safely say that the system is reactive as well. This is important information in itself, although node reactivity didn't prove to be a good approximation for total system reactivity. It needs at least the interaction between two nodes to provide a good measure of a lower bound.

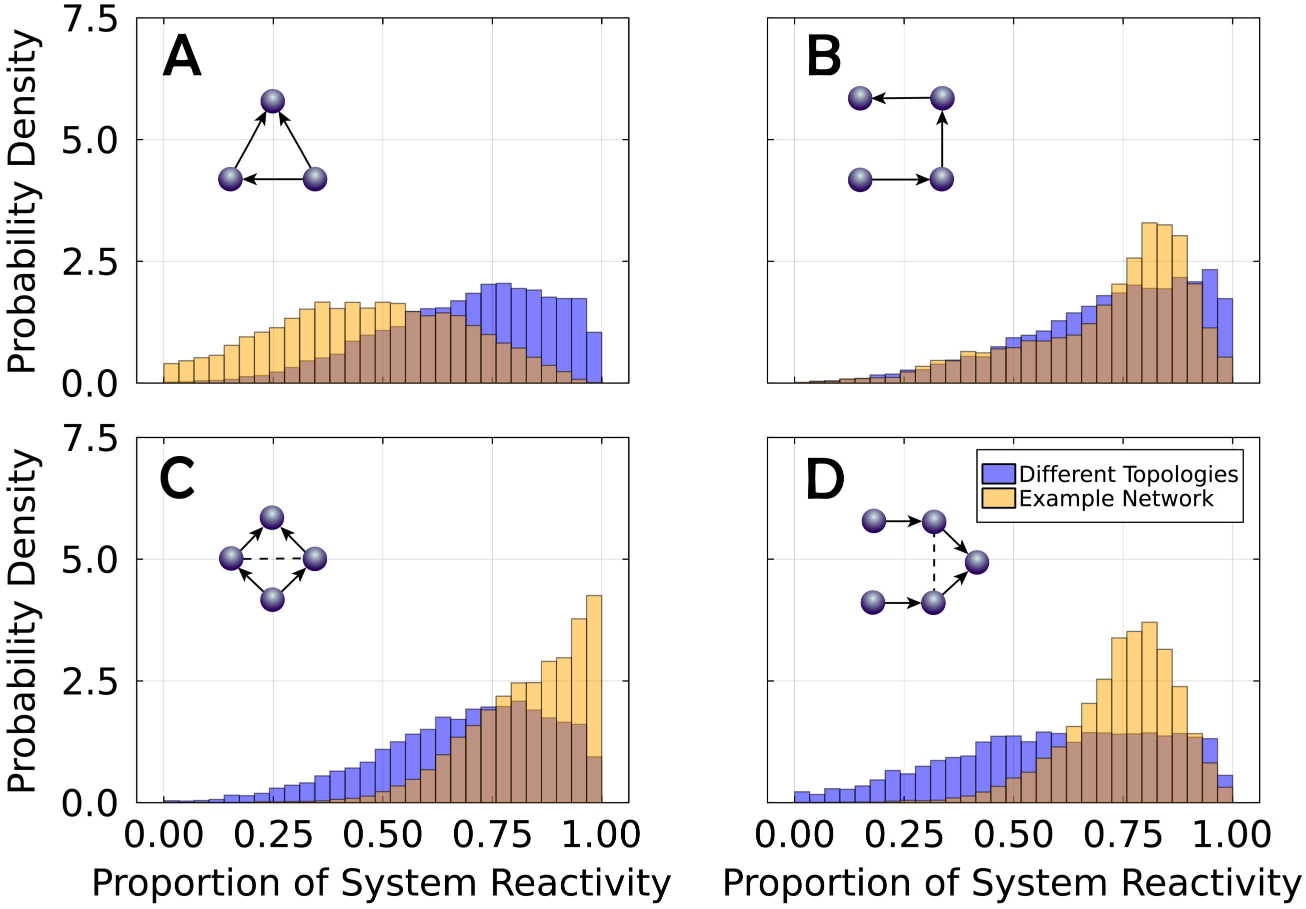

Fig. S1. Contribution of individual motifs to system reactivity for larger motifs. The histograms compare the proportion of total system reactivity explained by the most reactive instance of a single motif in 10^4 different communities. The motifs in question are the three-node omnivory motif (O, panel A), the tetra-trophic foodchain (FC, B), the diamond (D, C), and the combined tri-trophic foodchain (CTC). The structure of each motif is presented in the upper left corner. For the 15-node example network with a fixed topology (orange), the D motif accounts for the highest proportions of total system reactivity, reaching $\geq 85\%$ with a higher probability than any of the other shown motifs. In the set of networks with differing topologies (blue), O, FC, and D provide similar results with relatively strong contributions to system reactivity. The TCT motif was better suited to explain reactivity in the example network than it was in the case of the ensemble of networks, highlighting the importance of analysing network structure before deciding on a motif as a predictor for total system reactivity.

References

1. T Gross, U Feudel, Generalized models as a universal approach to the analysis of nonlinear dynamical systems. *Phys. Rev. E* **73**, 016205 (2006).
2. JC Massing, T Gross, Generalized modeling: A survey and guide (2021).
